# Transcriptomic Analysis of Alternative Splicing Events during Different Fruit Ripening Stages of *Coffea arabica* L.

**DOI:** 10.3390/genes15040459

**Published:** 2024-04-05

**Authors:** Haohao Yu, Xiaofei Bi, Zhongxian Li, Xingfei Fu, Yanan Li, Yaqi Li, Yang Yang, Dexin Liu, Guiping Li, Wenjiang Dong, Faguang Hu

**Affiliations:** 1 Institute of Tropical and Subtropical Cash Crops, Yunnan Academy of Agricultural Sciences, Baoshan 678000, China; y550570@163.com (H.Y.); rjsbxf@yaas.org.cn (X.B.);; 2 Spice and Beverage Research Institute, Chinese Academy of Tropical Agricultural Sciences, Wanning 571533, China

**Keywords:** *Coffea arabica* L., alternative splicing, fruit ripening, DSGs, DEGs

## Abstract

To date, genomic and transcriptomic data on *Coffea arabica* L. in public databases are very limited, and there has been no comprehensive integrated investigation conducted on alternative splicing (AS). Previously, we have constructed and sequenced eighteen RNA-seq libraries of *C. arabica* at different ripening stages of fruit development. From this dataset, a total of 3824, 2445, 2564, 2990, and 3162 DSGs were identified in a comparison of different fruit ripening stages. The largest proportion of DSGs, approximately 65%, were of the skipped exon (SE) type. Biologically, 9 and 29 differentially expressed DSGs in the spliceosome pathway and carbon metabolism pathway, respectively, were identified. These DSGs exhibited significant variations, primarily in S1 vs. S2 and S5 vs. S6, and they involve many aspects of organ development, hormone transduction, and the synthesis of flavor components. Through the examination of research findings regarding the biological functions and biochemical pathways associated with DSGs and DEGs, it was observed that six DSGs significantly enriched in ABC transporters, namely, *LOC113712394*, *LOC113726618*, *LOC113739972*, *LOC113725240*, *LOC113730214*, and *LOC113707447*, were continually down-regulated at the fruit ripening stage. In contrast, a total of four genes, which were *LOC113732777*, *LOC113727880*, *LOC113690566*, and *LOC113711936*, including those enriched in the cysteine and methionine metabolism, were continually up-regulated. Collectively, our findings may contribute to the exploration of alternative splicing mechanisms for focused investigations of potential genes associated with the ripening of fruits in *C. arabica*.

## 1. Introduction

Coffee, one of the most important cash crops worldwide, originated in Ethiopia and is classified under the *Coffea* genus within the Rubiaceae botanical family. It stands as the second largest raw material product in the world after petroleum. The *Coffea* genus encompasses over 124 species [1]. Of these, *C. arabica* and *C. canephora* are the predominant commercial coffee varieties, which collectively contribute to around 70% and 30% of global coffee production, respectively. The majority of coffee species are diploid (2*n* = 22), while *C. arabica* is an exception, as it is tetraploid (4*n* = 44), and it is attributed to a recent genome-wide duplication between *C. canephora* and *C. eugenioides* in the central Ethiopian plateaus [2,3]. This reflects an abundant genetic characterization of the *Coffea* genus, such as different agronomic traits [4], fruit quality [5,6], tolerance [7,8], and genome size [9].

The process of fruit ripening is a highly coordinated and intricate phenomenon characterized by dramatic alterations in fruit pigmentation, cell division and elongation, fruit softening, sugar and organic acid content, flavor, and aroma [10]. In coffee cultivars, the fruits usually show a uniform ripening process accompanied by a steady accumulation of dry matter and a gradual decrease in relative water content [11]. According to previous studies, the following three primary stages of coffee fruit development can be categorized according to color and metabolite changes: (i) the fruits have great hardness and turn color from green, accompanied by the synthesis of amino acids, nucleotides, and other components, (ii) an uneven red coloration of the fruit is present, (iii) a softening of fruits occurs with the accumulation of glycosides and secondary metabolites and the biosynthesis of aromas and flavors [12,13,14]. In recent years, the mechanisms of fruit ripening have been studied extensively at the transcriptional level, and the ripening regulatory network involves the regulation of master and downstream transcription factors (TFs) as well as DNA methylation. For instance, the dehydration-responsive element-binding *MaDREB* transcription factors serve as regulators during fruit ripening in banana [15]. The fusion of the *RIN-MC* gene, an active TF with repressor activity, is required for full fruit ripening [16]. Approximately 1% of the DNA methylome within the pericarp of the tomato fruit changes at the developmental stages [17]. Studies have shown that mRNA m^6^A methylation plays a role in regulating fruit ripening through distinct mechanisms [18]. The gene *SlDML2*, which encodes a DNA demethylase, affects the transcript level of *SlALKBH2*, establishing a feedback mechanism between DNA methylation and mRNA m^6^A methylation, ultimately controlling the fruit ripening in tomato [19]. In strawberries, the process of m^6^A methylation regulates its fruit ripening in a manner dependent on abscisic acid, which is distinct from what occurs in tomato [20]. 

Although there have been extensive studies on the regulation of gene transcription in developing fruit, the involvement of AS in the regulation of gene expression during this process remains poorly understood. AS serves as a crucial post-transcriptional mechanism for regulating gene expression. In eukaryotes, pre-mRNAs are transcribed in the nucleus and undergo AS events to form multiple mature mRNAs, which increase the complexity of the transcriptome and proteome [21]. Although their occurrence varies across plants and animals, understanding their evolution and adaptations across species remains key. Currently, five alternative splicing subtypes have been observed in plants [22], namely, skipped exon (SE), retained intron (RI), mutually exclusive exon (MXE), alternative 5′ splice sites (A5SS), and alternative 3’ splice sites (A3SS). Many studies on rice and *Arabidopsis* have consistently shown that RI presents the predominant subtype of AS, indicating that AS plays a vital role in regulating the growth and development in model plants [23,24]. In fact, AS is involved in all stages of plant growth and development, and it plays an important role in fruit development, flowering transition, and abiotic stress [25]. Previous studies have analyzed alternative splicing in fruit ripening and development, including tomato [26], blueberry [27], longan [28], banana [29], grape [30], Capsicum [31], kiwifruit [32], peach [33], sweet cherry [34], and watermelon [35]. However, different results have emerged. For instance, there was a notable increase in the occurrence of AS at the ripe stage compared to the unripe stage in cucumber and melon. Conversely, papaya and peach exhibited the opposite trend, suggesting that distinct AS strategies are employed by different developmental stages to fulfill their specific functions [36].

For coffee, the entire production process from seed to cup goes through growth and development, harvesting, and processing before it reaches the market and consumers. Coffee fruit development plays a key role in the final formation of flavorful, high-quality roasted coffee beans. In our previous studies, we screened some of the genes that regulate coffee fruit ripening and clarified the synthesis of green bean substances at various fruit ripening stages [37]. However, the AS events of coffee during fruit ripening remain unknown. The study of AS events contributes to comprehension of the mechanism of coffee fruit ripening and development, providing a better representation of the dynamic changes that occur during fruit ripening at the post-transcriptional stage. In this study, RNA-seq was employed to examine AS events in relation to six different ripening stages within *C. arabica*. The detailed ripening divisions were able to closely track the changes in AS events during fruit development. To further understand the genetic regulation of fruit development, we compared and screened AS events in different ripening stages and linked them to biological process. Furthermore, we statistically analyzed genes that were both spliced and differentially expressed. In conclusion, the aim of this study was to analyze AS patterns during coffee fruit development, aiming to provide a basis for exploring the alternative splicing mechanism with the mining of potential splicing factors, especially in the absence of relevant studies on *Coffea* spp.

## 2. Materials and Methods

### 2.1. Plant Materials

The test material employed in this study was the *C. arabica* cultivar *Catimor*. Six fruit-colored (S1, green color; S2, yellowish-green color; S3, reddish-green color; S4, reddish-orange color; S5, fully red color; S6, purple-red color) coffee plants of the same cultivar were chosen for this study, with a minimum of twenty plants for each fruit color being planted in four rows. The plants were spaced 1 m apart within rows and 2 m apart between rows. Six random plants were selected for sampling, and six fully mature and evenly colored fresh cherries were harvested from each color category. Each harvested fruit was carefully labeled and promptly placed in liquid nitrogen for preservation. Subsequently, all the samples were then stored at −80 °C in an ultra-low-temperature refrigerator. All the samples used in this study were the same as those used in our previous study [38].

### 2.2. RNA-seq

Total RNA was isolated from eighteen samples (from six stages and three replicates) with a Sigma Spectrum Plant Total RNA Kit (Sigma-Aldrich, St. Louis, MO, USA) according to the manufacturer’s standard protocol. An RNase-free DNase I kit (TaKaRa, Kyoto, Japan) was used to remove genomic DNA and obtain high-quality RNA. The RNA concentration was accurately measured with a Qubit 2.0 fluorescence meter. The RNA quality was assessed using an Agilent 2100 Bioanalyzer (Agilent Technologies, Santa Clara, CA, USA) and checked using RNase-free agarose gel electrophoresis. mRNA was enriched by Oligo (dT) beads (Epicentre, Madison, WI, USA). In addition, mRNA was purified using an AMPure XP system (Beckman Coulter, Brea, CA, USA). The product was quantified by Agilent high-sensitivity DNA analysis on a Bioanalyzer 2100 system (Agilent Technologies, Santa Clara, CA, USA). The cDNA libraries were then performed on the Illumina HiSeq2000 platform (each read of sequenced fragment was 150 bp) by MetWare Biotechnology Co., Ltd. (Wuhan, China).

### 2.3. Reads Mapping and Transcript Assembly

The Fastp software (v0.19.3) was used to obtain high-quality sequenced reads. All clean reads from the sequenced sample were mapped to the reference genome of *C. arabica* (GCF_003713225.1_Cara_1.0_genomic.fna) by HISAT2. The application of Stringtie was used to assemble the mapped reads into transcripts. The reads mapped onto the annotated gene regions were subjected to the statistical analysis. The expression level of each gene was normalized to the FPKM value.

### 2.4. Identification of Differential Alternative Splicing Events and Differentially Expressed Genes

For the AS analysis, the rMATS software v4.0.2 (https://rnaseq-mats.sourceforge.io/, accessed on 7 January 2024) was used to compare replicate biological samples representing different ripening stages of *C. arabica*. We identified AS events with a false discovery rate (FDR) < 0.05 in comparison to AS events, enabling the delineation of DAS events between different ripeness levels of the coffee fruit. The classification of AS events was as follows: SE, skipped exon; MXE, mutually exclusive exon; A5SS, alternative 5′ splice site; A3SS, alternative 3′ splice site; and RI, retained intron. Similarly, the RNA-seq data underwent differential expression analysis between two distinct groups through the utilization of the DESeq2 software (v1.22.1). Differentially expressed genes (DEGs) were identified through statistical analysis using a *t*-test (corrected by false discovery rate, FDR). Probe-sets with an FDR-corrected *p*-value < 0.05 and log_2_ |fold change| > 1 were considered as differentially expressed genes/transcripts.

### 2.5. Functional Enrichment Analysis

Trend analysis was performed using the STEM software (v1.3.11), and FPKM values were normalized by log_2_. The Gene Ontology (GO) and Kyoto Encyclopedia of Genes and Genomes (KEGG) databases were used to construct the functional cluster of the DSGs or DEGs. The calculation of the *p*-value was performed with an FDR correction, taking an FDR < 0.05 as a threshold. Fisher’s exact test (*p*-value < 0.05) was utilized to identify significantly enriched KEGG pathways in comparison to the entire genome background.

### 2.6. qRT-PCR Validation of AS Events

The expression of two genes of 828 overlapping genes between the DEGs and DSGs was analyzed by quantitative real-time PCR (qRT-PCR) to validate the RNA-Seq data. For the qRT-PCR analysis, the specific primers for the target gene were prepared with Oligo 7. The reaction mixture was formulated using a SYBR Green PCR Kit (QIAGEN, Dusseldorf, Germany) by following the manufacturer’s protocol. The actin7 gene served as the reference gene. The qRT–PCR protocol and relative gene expression analysis were performed as described in a previous study.

### 2.7. Data Presentation

All the statistical analyses and plots were carried out using GraphPad Prism 8 (GraphPad Software Inc., San Diego, CA, USA) and R packages (version 3.3.2).

## 3. Results

### 3.1. Basic Information on Transcripts

Eighteen RNA-seq libraries were used to investigate the regulation of AS in coffee fruits at different ripening stages. These libraries consisted of six stages of fruit ripening: green fruit (S1), yellowish-green fruit (S2), reddish-green fruit (S3), reddish-orange fruit (S4), fully red fruit (S5), and purplish-red fruit (S6). Each sample was sequenced separately, and three biological replicates were performed. Our previous study revealed the differential expression of thousands of genes in coffee fruits at different ripening stages [38]. Here, we used the same RNA-seq dataset to further examine the comprehensive profiles of AS events. In total, the transcriptomic analysis yielded an average read count of 48,769,917 bp (ranging from 44,237,776 to 58,459,260 bp) of the raw reads. After trimming and modification, it was calculated that the mean value of the high-quality clean reads was 45,590,061 bp (ranging from 40,122,252 to 55,778,906 bp) with a GC content of 43.36% (ranging from 41.11% to 43.89%). The modified sequences were then aligned with the reference genome of C. arabica in the NCBI database, which resulted in an overall alignment of all samples ranging from 91.62% to 93.88% (Table S1). Therefore, the high quality of the assembled RNA-seq data could be used for the detection and analysis of AS events, which is consistent with previous reports.

### 3.2. Identification and Classification of Alternative Splicing Events

**Figure 1:**
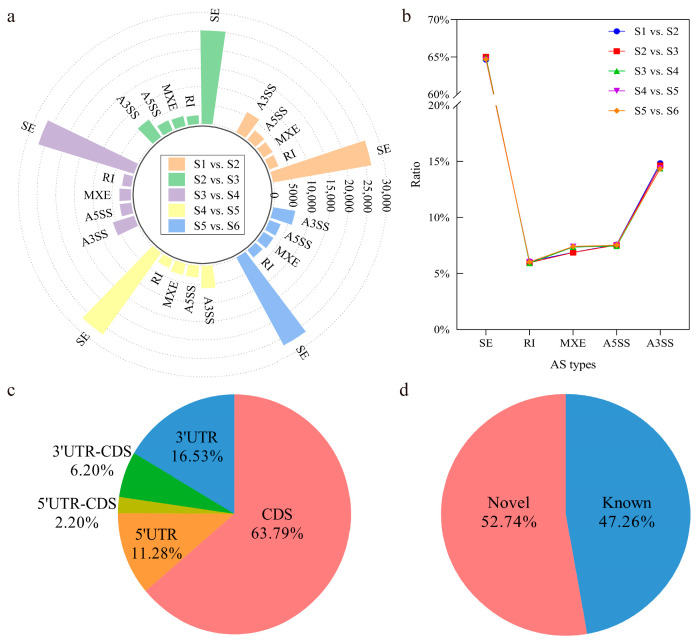
Summary of alternative splicing events during different ripening stages of *C. arabica*. (**a**) Categories and numbers of different types of alternative splicing events; (**b**) proportion of each alternative splicing type; (**c**) distribution of alternative splice events in the annotated loci; (**d**) distribution of alternative splice events levels.

RNA-seq data were used to investigate the occurrence of AS events in coffee fruits at different ripening stages. Briefly, the alignment of the clean reads with the reference genome of *C. arabica* was performed, followed by identification via the rMATS tool. Among the assembled transcripts, five types of AS events were identified, including skipped exon (SE), retained intron (RI), mutually exclusive exon (MXE), alternative 5′ splice sites (A5SS), and alternative 3′ splice sites (A3SS). A total of 203,986 alternative splicing events were identified in all the samples, indicating their possible ability to regulate the growth and fruit ripening of *C. arabica* (Table S2). The number of each type of AS event is shown in Figure 1a. SE was the most frequent AS type, with a total of 132132 AS events (64.78%) in the six fruit ripening stages, followed by A3SS (29,612, 14.52%), A5SS (15,342, 7.52%), and MXE (14,659, 7.19%), and RI was the rarest (12,241, 6.00%) (Figure 1b). In general, there was little difference in the number of AS events at the different ripening stages.

Most alternative splicing events were located in the coding regions (CDS), with a proportion of 63.79% among the isoforms in the annotated loci, followed by 16.53% in the 3′ UTR, 11.28% in the 5′ UTR, 6.20% in the 3′ UTR-CDS, and 2.20% in the 5′ UTR-CDS (Figure 1c). The results indicated that the CDS was strongly influenced by AS. Among the AS events, 47.26% and 52.74% were known splicing events and novel splicing events, respectively (Figure 1d). The results suggested that the form of the transcripts varied greatly during the ripening stages of the coffee fruit.

### 3.3. Dynamic Characterization and Function Analysis of Alternative Splicing Events

**Figure 2:**
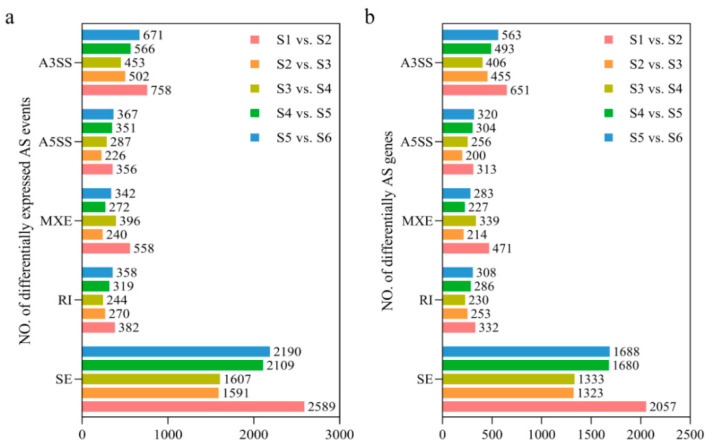
Differential alternative splicing events (**a**) and differentially spliced genes (**b**) during different ripening stages of *C. arabica*.

To investigate the variation in the types of AS events during the different ripening stages of *C. arabica*, we screened the number of AS events among the five groups by an FDR (false discovery rate from *p*-value) < 0.05. Then, the specific numbers of differential alternative splicing (DAS) events and differentially spliced genes (DSGs) were obtained. As shown in Figure 2, a total of 4643, 2829, 2987, 3617, and 3928 DAS events were identified for S1 vs. S2, S2 vs. S3, S3 vs. S4, S4 vs. S5, and S5 vs. S6, respectively. Furthermore, each splicing type had a different number of AS types. Among all the types of AS events, SE was the most abundantly counted AS type under the S1 vs. S2 (2589), S2 vs. S3 (1591), S3 vs. S4 (1607), S4 vs. S5 (2109), and S5 vs. S6 (2190) phases. Overall, the number of DAS events was higher in S1 vs. S2 and S5 vs. S6. These two phases correspond to the expansion growth phase of green fruit and the accumulation of secondary metabolites and sugars in fully red fruit, respectively. The same trend was observed in the DSGs (Figure 2).

**Figure 3:**
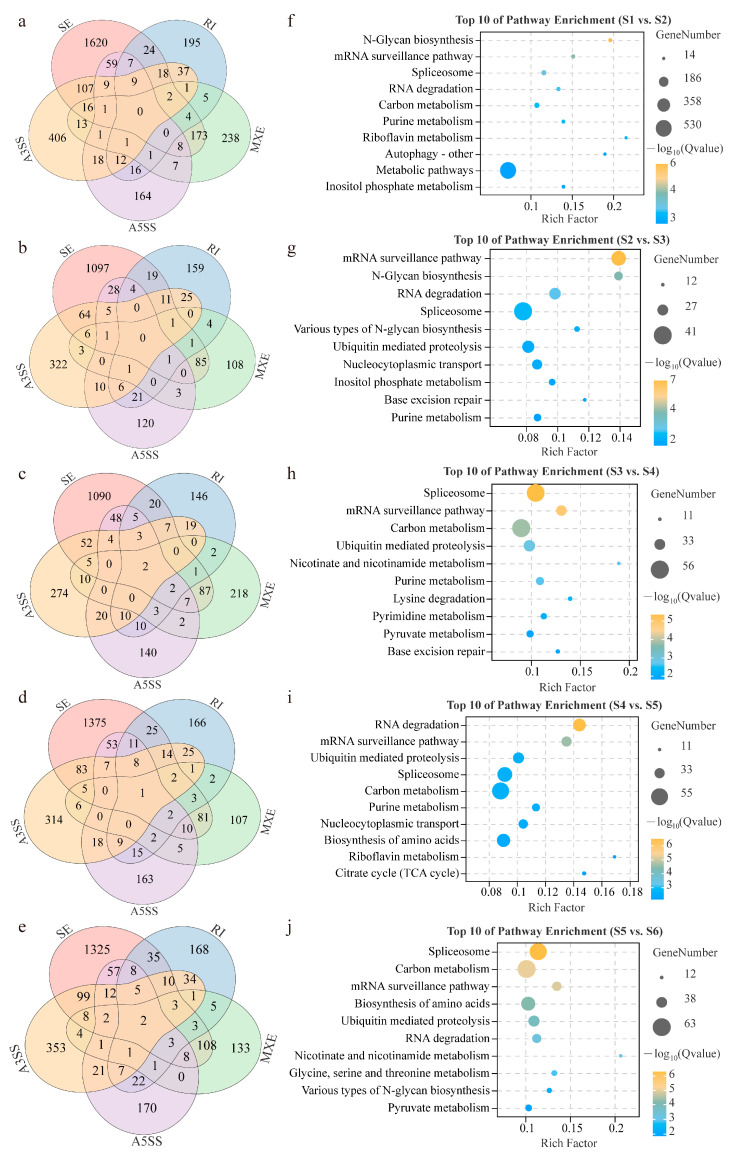
Differentially spliced genes (DSGs) and related pathways identified in different ripening stages of *C. arabica*. (**a**–**e**) Venn diagram of S1 vs. S2, S2 vs. S3, S3 vs. S4, S4 vs. S5, and S5 vs. S6, respectively; (**f**–**j**) KEGG enrichment analysis of S1 vs. S2, S2 vs. S3, S3 vs. S4, S4 vs. S5, and S5 vs. S6, respectively.

Then, we analyzed splicing at the different ripening stages based on the obtained DEGs. Among all the AS genes in the whole fruit ripening period, the largest fraction of DSGs were associated with the splicing of SE, whereas the smallest fraction of DSGs were associated with AS genes with RI and A5SS. Most AS genes had only one type of AS in the different ripening stages (Figure 3a–e and Table S3). For instance, a total of 1620, 1097, 1090, 1375, and 1325 DSGs exhibited unique SE-type AS events at the five fruit ripening stages, respectively. Among the DSGs, only four genes overlapped with five AS types (*LOC113707757* exhibited five AS types in both S3 vs. S4 and S4 vs. S5). Among the overlapping genes, *LOC113732495*, *LOC113736605*, *LOC113707757*, and *LOC113697369* share sequence similarity with GRF2 (general regulatory factor 2), CTR1 (kinase superfamily protein), ELF7 (hydroxyproline-rich glycoprotein family protein), and STT3A (staurosporin and temperature-sensitive 3-like A) in *Arabidopsis* and may play important roles in fruit ripening, development, or component accumulation (Table S4).

To further clarify the biological pathways involved in the significant enrichment of DSGs during fruit ripening, we performed a KEGG enrichment analysis. According to the KEGG annotations, 10 pathways were significantly enriched in total for the five groups (q-value < 0.05). As shown in Figure 3f, the representative enriched terms were “N-glycan biosynthesis”, “mRNA surveillance pathway”, and “spliceosome” in the S1 vs. S2 stage. For the S2 vs. S3 stage, the representative enriched terms were “mRNA surveillance pathway”, “N-glycan biosynthesis”, and “RNA degradation” (Figure 3g). For the S3 vs. S4 stage, the representative enriched terms were “spliceosome”, “mRNA surveillance pathway”, and “carbon metabolism” (Figure 3h). For the S4 vs. S5 stage, the representative enriched terms were “RNA degradation”, “mRNA surveillance pathway”, and “ubiquitin-mediated proteolysis” (Figure 3i). For the S5 vs. S6 stage, the representative enriched terms were “spliceosome”, “carbon metabolism”, and “mRNA surveillance pathway” (Figure 3j). In general, “spliceosome”, “carbon metabolism”, “mRNA surveillance pathway”, “RNA degradation”, and “N-glycan biosynthesis” were the most frequent pathways of DSGs in the whole ripening stage of coffee fruit. The genes of these pathways undergo continuous alternative splicing events for post-transcriptional modification throughout the developmental period of coffee fruit ripening and perform a variety of biological functions (Table S5).

### 3.4. Comparative Analysis of DSGs and DEGs

**Figure 4:**
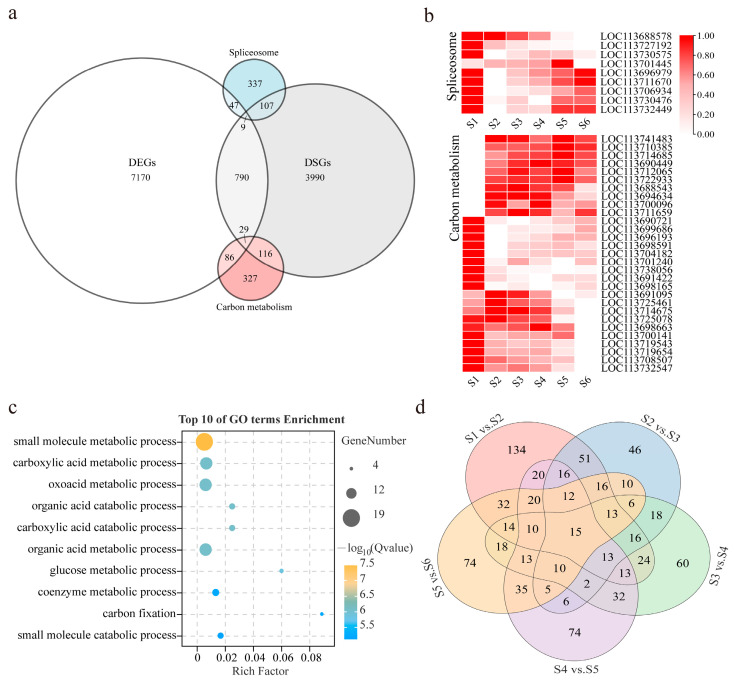
Differentially spliced genes (DSGs) and differentially expressed genes (DEGs), and related pathways identified in the whole ripening stages of *C. arabica*. (**a**) Venn diagram shows overlap of genes with DSGs, DEGs, and gene-related spliceosome and carbon metabolism pathways; (**b**) heat map of overlapped genes between DEGs, DSGs, and spliceosome and carbon metabolism pathways, from low (white) to high (red); (**c**) the top ten enriched GO terms of genes between DEGs, DSGs, and spliceosome and carbon metabolism pathways; (**d**) Venn diagram showing AS events of overlapped genes between DSGs and DEGs at different ripening stages.

In a previous study, we identified a total of 8131 genes that were significantly differentially expressed throughout the whole fruit ripening stage. To explore how many genes with DAS events occur in differentially expressed genes (DEGs), a comparison was made among the DEGs, genes with DAS events, and genes in the two biological pathways (“spliceosome”, and “carbon metabolism”) that were significantly enriched and most abundant in the DSGs throughout the whole fruit ripening stage (Figure 3f–j and Table S5). We found that 828 genes with DAS events were also differentially expressed, of which 9 were spliceosome-related genes and 29 were carbon-metabolism-related genes according to KEGG enrichment analysis. Nevertheless, the majority of the DSGs (4213) were not differentially expressed (Figure 4a). Then, we identified these 9 and 29 DEGs that underwent DAS events in the “spliceosome” and “carbon metabolism” pathways, respectively. The heatmap shows that these genes were mainly differentially expressed in S1 vs. S2 and S5 vs. S6 (Figure 4b). GO functional analysis revealed that these genes were mainly enriched in the “small-molecule metabolic process”, “carboxylic acid metabolic process”, “oxoacid metabolic process”, etc., (Figure 4c and Table S6), suggesting that biological process and metabolic activity are more intense during the expansion and growth phase of green fruit (S1 vs. S2) and the accumulation of secondary metabolites and sugars in fully red fruit (S5 vs. S6). Subsequently, we plotted the 828 DEGs with DAS events into a Venn diagram using the five comparison settings, including S1 vs. S2, S2 vs. S3, S3 vs. S4, S4 vs. S5, and S5 vs. S6 (Figure 4d). A total of 388 DEGs that underwent DAS events were specific to one ripening stage, and 15 genes underwent simultaneous differential expression and DAS events at all five ripening stages.

**Figure 5:**
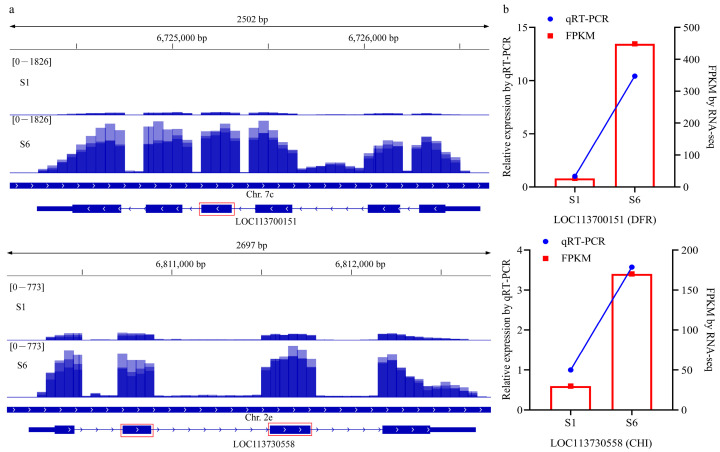
Validation of randomly selected DSGs. (**a**) The IGV (Integrated Genomics Viewer) images and gene structures of the DFR (*LOC113700151*) and CHI (*LOC113730558*). The red boxes over the IGV images and the gene structures represent the location where alternative splicing events occurred; (**b**) validation of the DFR (*LOC113700151*) and CHI (*LOC113730558*) using qRT-PCR.

To verify the DAS events and gene expression by RNA-seq, two genes were randomly selected in the 828 candidate DSGs for further validation. The expression of these two genes in the two fruit ripening stages of *C. arabica* was investigated in this study (Figure 5a). Next, we validated the skipped exon event of DFR (*LOC113700151*) and CHI (*LOC113730558*) by qRT–PCR. Figure 5b shows significant agreement between the expression patterns obtained by the RNA-Seq and qRT–PCR methods.

### 3.5. Biological Function Analysis between DSGs and DEGs

**Figure 6:**
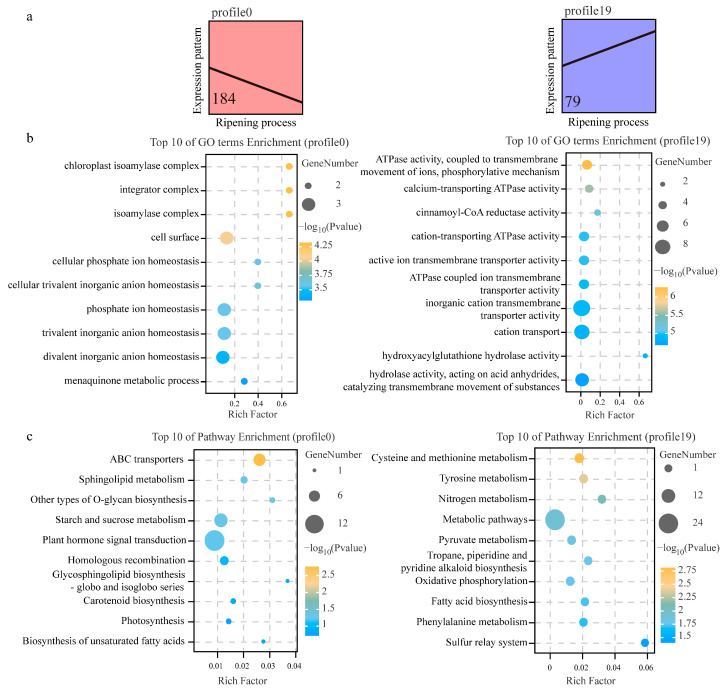
Functional enrichment analysis of DSGs in significant expression trends of profile 0 and profile 19. (**a**) Significant expression trends of profile 0 and profile 19 (with *p* < 0.05) at different ripening stages; (**b**) GO enrichment analysis of profile 0 and profile 19; (**c**) KEGG analysis of profile 0 and profile 19.

The gene expression trends of 828 differentially expressed DSGs at different ripening stages of *C. arabica* were analyzed, and a total of 20 trend profiles were obtained (Figure S1). According to the test of significance for the gene expression trend analysis (*p* < 0.05), two target trend profiles, including continually decreasing expression (profile 0) and continually increasing expression (profile 19), were selected for biological function analysis, among which 184 and 79 genes were in profile 0 and profile 19, respectively (Figure S1 and Figure 6a).

**Figure 7:**
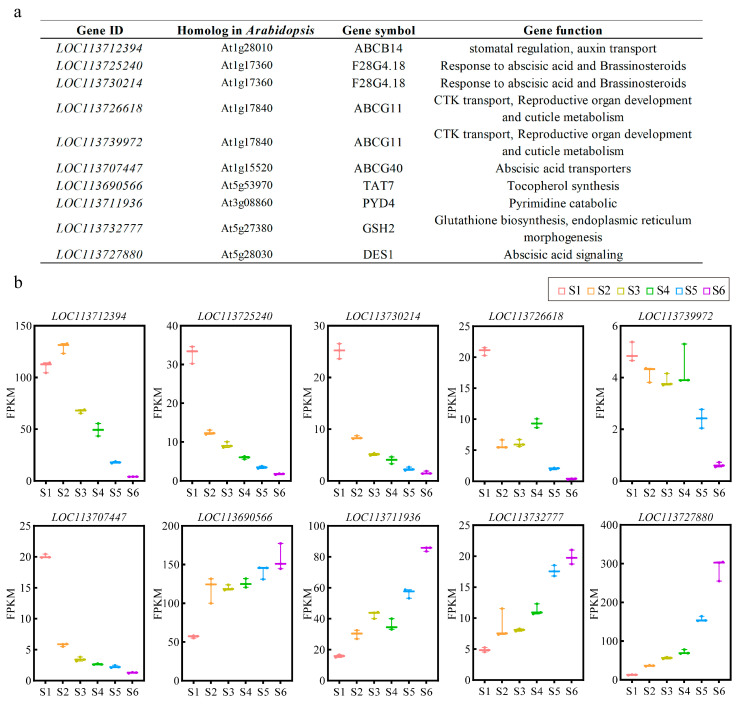
Identification of ABC transporters and cysteine and methionine metabolic components associated with candidate DSGs. (**a**) List of candidate DSGs; (**b**) expression of candidate DSGs.

To investigate the biological functions of the candidate DSGs with different expression trends, Gene Ontology (GO) and Kyoto Encyclopedia of Genes and Genomes (KEGG) enrichment analysis were performed. The GO analysis revealed that “chloroplast isoamylase complex”, “integrator complex”, and “isoamylase complex” were the most enriched in profile 0, and “ATP activity, coupled to transmembrane movement of ions, phosphorylative mechanism”, “calcium-transporting ATPase activity”, and “cinnamoyl-CoA reductase activity” were the most enriched in profile 19 (Figure 6b). This suggests that DSGs with consistently decreasing expression are mainly enriched mainly in cellular components associated with enzymatic activities, while DSGs with consistently increasing expression are mainly enriched in molecular functions related to transmembrane transport. In addition, the KEGG analysis indicated that “ABC transporters” and “cysteine and methionine metabolism” were the top-ranked pathways in profile 0 and profile 19, respectively (Figure 6c). Further analysis revealed that the *Arabidopsis* homologues of the ABC transporters and cysteine and methionine metabolism components enriched in the candidate DSGs are involved in hormonal responses, nutrient metabolism, and organ development (Figure 7a). The expression of these candidate DSGs is shown in Figure 7b, where ABC-transporter-related DSGs had lower expression after coffee fruit ripening, while cysteine- and methionine-metabolism-related DSGs showed the opposite trend.

## 4. Discussion

AS is a common post-transcriptional regulatory mechanism in higher eukaryotes that helps to increase the complexity of gene expression and the diversity of protein functions, thus enhancing the adaptability of organisms [39]. Our study aimed to identify AS events during fruit ripening in *C. arabica*, and six ripening stages, from green to purplish-red fruits, were investigated. We found that genes underwent continuous AS events throughout the ripening of coffee fruit, resulting in a significant increase in transcriptome diversity. Approximately equal numbers of the observed AS events were found in five AS types under all ripening stages. Nearly 65% of the DSGs of coffee fruit underwent a skipped exon, a much greater frequency than that of other AS types (Figure 1b). These finding differ from the results of some fruit ripening studies. For instance, IR was the most common AS type in cucumber, melon, and papaya, while A3SS was the most common AS type in peach [35]. For grape berries, the predominant AS type varies across studies [31,40,41], which may be caused by the different varieties, ripening stages, or genomes used. Indeed, comprehensive information on AS transcripts in *Arabidopsis* indicates that many low-abundance and incompletely spliced RI transcripts constitute noise in the splicing process, suggesting that the number of RIs may be overestimated in the genome [42].

Fruit formation in *C. arabica* is attributed to morphological changes, pigment accumulation, and compositional changes accompanied by intense metabolic activity [13]. In this study, AS events were more abundant at the S1 vs. S2 stage during the coffee fruit ripening stages, indicating that AS is more frequently regulated at the early stage of fruit development. Previous studies have shown that the number of AS events in fruits such as watermelon and kiwifruit is significantly increased in the ripe stage than in the unripe stage [36,43], while the opposite trend has been shown in papaya and peach. This may be related to species characteristics. Coffee fruits begin to expand and turn color during the early growth stages, implying that different species adopt different AS strategies for their specific functions during developmental stages. In addition, the DAS events detected in this study composed only a fraction (approximately 8.83%) of the total number of all AS events retained in each comparison, reflecting that AS events mostly occurred without being regulated. These AS events were relatively conserved and necessary for the ripening stage of coffee fruit.

A total of 3824, 2445, 2564, 2990, and 3162 DSGs with significant splicing differences were identified in the five comparison groups in this study, and the most enriched and abundant pathways in the DSGs were spliceosome and carbon metabolism. We identified 9 and 29 differentially expressed DSGs associated with the spliceosome and carbon metabolism, respectively (Figure 4). Splicing factors are essential for regulating AS during plant development. For instance, the *Arabidopsis* homolog of *LOC113701445*, ALY RNA-binding proteins, are required for nucleocytosolic mRNA translocation and the regulation of plant growth and development, and they are differentially expressed in reproductive tissue [44]. The developmentally regulated expression of the *Arabidopsis* homolog of *LOC113688578*, heat-inducible Hsp70, in mature dry seeds and roots suggests prominent roles in seed maturation and root growth [45]. Additionally, four genes encoding Ser-/Arg-rich proteins involved in pre-mRNA splicing were found to generate AS events during fruit ripening in this study, including *LOC113730476* (a homolog of SCL30), *LOC113696979* (a homolog of SCL30a), *LOC113706934* (a homolog of RSZ22a), and *LOC113730575* (a homolog of RS2Z33). Interactions between Ser-/Arg-rich proteins and pre-mRNAs are essential for alternative splicing and play a critical role in ABA and sugar signaling as well as in the regulation of seed development [46,47].

Many studies have reported changes in carbohydrate content during fruit development and ripening, as carbohydrate accumulation affects fruit quality at harvest, including flavor, aroma, and softening [48,49]. In coffee, ripe coffee fruits have relatively higher concentrations of carbohydrates, which increase coffee sweetness and also act as aroma precursors to increase pyrazine, furan, and ketone contents after roasting to increase the aroma of the coffee [50,51]. Our data indicate that 10 DSGs involved in carbon metabolism, encoding the PPC3 (*LOC113688543*), ACAT2 (*LOC113690449*), clCDH (*LOC113700096*), PSP (*LOC113710385*), G6PD6 (*LOC113722933*), SHM1 (*LOC113712065*, *LOC113714685*), single hybrid motif superfamily protein (*LOC113741483*), phosphoglycerate mutase family protein (*LOC113711659*), and CaLB domain family protein (*LOC113694634*), were significantly up-regulated during early fruit development. These DSGs play a vital role in promoting plant growth and development, maintaining redox homeostasis in seeds, and regulating photorespiration. We also identified several carbon-metabolism-related DSGs that were significantly down-regulated during early fruit development, such as NADP-ME2 (*LOC113691422*). The increase in NADP-ME2 expression has profound consequences for the modulation of primary metabolism in *Arabidopsis*, resulting in reduced root size, delayed flowering time, and redox imbalance [52]. Furthermore, we observed a sustained down-regulation of the PCK1-encoding gene *LOC113714675* and the FDH-encoding gene *LOC113708507* during fruit ripening. PCK1 is mainly involved in the gluconeogenesis pathway, and the overexpression of the PCK1 gene impairs the accumulation of soluble sugars [53], while FDH is mainly involved in the oxidation of mesoformate. Thus, the down-regulation of *LOC113714675* and *LOC113708507* in coffee fruit may be aimed at increasing sweetness and acidity.

Further analysis revealed that some DSGs, such as ABCB14 (*LOC113712394*), ABCG11 (*LOC113726618*, *LOC113739972*), F28G4.18 (*LOC113725240*, *LOC113730214*), and ABCG40 (*LOC113707447*), were enriched in the ABC transporters (biosynthesis of the ATP-binding cassette transporter), which belong to a superfamily of oligopeptides responsible for transporting a variety of substrates across the membrane. These ABC proteins are involved in a variety of functions in plants, including cell division, nutrient uptake, hormone transduction, and stomatal regulation [54,55,56,57,58,59]. Therefore, these genes may have similar functions in *C. arabica*. It was shown that an ABC protein, ABCC1, localizes to the tonoplast and is involved in the transport of glucosylated anthocyanidins [60]. Thus, as a result of our previous study, a down-regulated expression of DSGs involved in ABC transporters may disrupt the accumulation of certain anthocyanin metabolites. Then, we identified four DSGs enriched in cysteine and methionine metabolism, including GSH2 (*LOC113732777*), DES1 (*LOC113727880*), TAT7 (*LOC113690566*), and PYD4 (*LOC113711936*). GSH is a non-enzymatic antioxidant that plays an important role in plant growth and various developmental processes, ranging from developmental aspects to various stress responses [61]. It is worth noting that according to research, the up-regulated expression of glutathione-metabolism-related genes (GSH1, GSH2, GSTF1, and GSTF5) is involved in the regulation of tomato fruit ripening [62]. GSH can also co-transport with anthocyanins, which participate in the anthocyanin metabolism pathway. DES1 is hormonally regulated, and an increase in DES1 expression in response to ABA was recently demonstrated [63]. Accordingly, the DES1-encoding gene *LOC113727880* was consistently up-regulated during the fruit ripening stage, possibly in combination with the increase in ABA content. TAT7 and PYD4 are involved in the metabolic processes of tyrosine and alanine, respectively. The ripening of Capsicum annuum fruit is characterized by an increase in tyrosine nitration, whereas alanine has been reported to accumulate during tomato fruit ripening [64,65]. Thus, the up-regulated expression of the TAT7-encoding gene *LOC113690566* and PYD4-encoding gene *LOC113711936* regulate the accumulation of amino acids.

## 5. Conclusions

We report here, for the first time, a precise analysis of AS regulation during coffee fruit ripening (the six ripening stages from green to purple-red fruit). Based on transcriptome analysis, the results of this study revealed the dynamic changes in AS and its possible regulatory functions during coffee fruit ripening. The results showed that SE was the predominant splicing type in coffee fruit ripening. The statistical analysis identified 3824, 2445, 2564, 2990, and 3162 DSGs at five comparison groups, respectively, with more abundant AS events at the early stage of fruit development, indicating its frequent regulation. Based on the biological analysis of these DSGs, we propose that changes in the abundance and expression levels of AS transcripts may influence spliceosome and carbon metabolism to promote fruit ripening. In addition, a systemic approach was used to understand and dissect the functions of DEGs and DSGs during fruit ripening and AS events in ABC-transporter-related genes and cysteine- and methionine-metabolism-related genes, as well as their expression levels. A total of 10 candidate DSGs were identified in these two biological pathways, both of which act via the direct or indirect regulation mode to modulate coffee fruit ripening. This finding implies that the modulation of AS by ABC transporters and cysteine and methionine metabolism are candidate strategies for regulating fruit ripening. The role of the many genes found to undergo AS events in this study has been poorly described in plants, and the functions of the different splice isoforms are even less well understood. Further characterization of these genes will certainly be useful for improving our understanding of the process of fruit ripening. Moreover, the coffee genotypes selected in this study are widely planted in China with a good yield and resistance, as well as with an ordinary quality and low price. With the increase in coffee consumption in China, it is imperative to grow high-quality coffee genotypes. Further studies should therefore provide perspectives for the selection of high-quality genotypes.

## Data Availability

Data will be made available on request.

## Supplementary Materials

The following supporting information can be downloaded at: https://www.mdpi.com/article/10.3390/genes15040459/s1, Figure S1: Trend analysis of overlapping genes between DSGs and DEGs in coffee fruit at six ripening stages. Block diagrams with colors indicate trend profiles with a significance level under the cutoff for statistical analysis (*p* < 0.05). The numbers in the blocks indicate counts of genes in the trend profiles; Table S1: Basic information—alignment; Table S2: Comprehensive statistics for all AS events under different ripening stages of coffee fruits; Table S3: The numbers of AS genes with different types of alternative splicing at different ripening stages of *Coffea arabica* L.; Table S4: List of overlapped genes with five AS types occurring; Table S5: List of genes in the five biological pathways most enriched for AS; Table S6: List of genes that are both differentially spliced and differentially expressed in the spliceosome and carbon metabolic pathways.
