# Supplementary material for: Transcriptomic Analysis of Alternative Splicing Events during Different Fruit Ripening Stages of Coffea arabica L
Source: Genes (Basel). 2024 Apr 5;15(4):459. doi: 10.3390/genes15040459 (PMC11050144; doi:10.3390/genes15040459)

Figure S1: Trend analysis of overlapping genes between DSGs and DEGs in coffee fruit at six ripening stages. Block diagrams with colors indicate trend profiles with a significance level under the cutoff for statistical analysis (P < 0.05). The numbers in the blocks indicate counts of genes in the trend profiles.

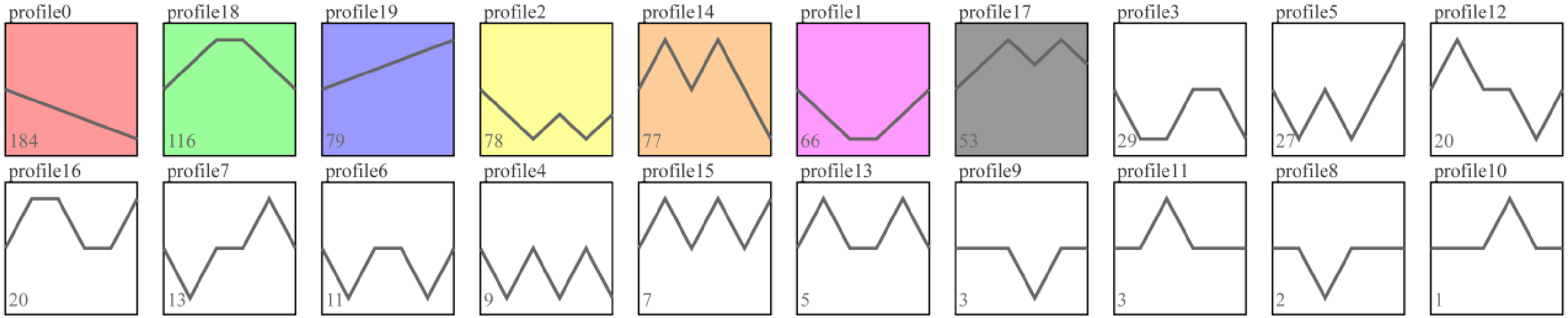

Supplement: Supplementary file 1 [file genes-15-00459-s001.zip › FigureS1.pdf]
